# ISAR Imaging of Ship Targets Based on an Integrated Cubic Phase Bilinear Autocorrelation Function

**DOI:** 10.3390/s17030498

**Published:** 2017-03-03

**Authors:** Jibin Zheng, Hongwei Liu, Zheng Liu, Qing Huo Liu

**Affiliations:** 1 National Laboratory of Radar Signal Processing, Xidian University, Xi’an 710071, China; hwliu@xidian.edu.cn (H.L.); lz@xidian.edu.cn (Z.L.); 2 Collaborative Innovation Center of Information Sensing and Understanding, Xidian University, Xi’an 710071, China; 3 Department of Electronic Engineering, Duke University, Durham, NC 27708, USA; qhliu@ee.duke.edu

**Keywords:** inverse synthetic aperture radar imaging, cubic phase signal, range-instantaneous-Doppler technique

## Abstract

For inverse synthetic aperture radar (ISAR) imaging of a ship target moving with ocean waves, the image constructed with the standard range-Doppler (RD) technique is blurred and the range-instantaneous-Doppler (RID) technique has to be used to improve the image quality. In this paper, azimuth echoes in a range cell of the ship target are modeled as noisy multicomponent cubic phase signals (CPSs) after the motion compensation and a RID ISAR imaging algorithm is proposed based on the integrated cubic phase bilinear autocorrelation function (ICPBAF). The ICPBAF is bilinear and based on the two-dimensionally coherent energy accumulation. Compared to five other estimation algorithms, the ICPBAF can acquire higher cross term suppression and anti-noise performance with a reasonable computational cost. Through simulations and analyses with the synthetic model and real radar data, we verify the effectiveness of the ICPBAF and corresponding RID ISAR imaging algorithm.

## 1. Introduction

High-resolution inverse synthetic aperture radar (ISAR) imaging has attracted the attention of radar researchers in the past three decades due to its importance in civil and military applications [1,2,3,4]. Two challenges of the high-resolution ISAR imaging are the motion compensation and scatterer-dependent Doppler spread compensation [3,5,6,7]. The motion compensation includes the translational range migration compensation, migration through resolution cells compensation and phase focusing [5,6,7]. The scatterer-dependent Doppler spread is corresponding to the coordinate of the scatterer, and the range-instantaneous-Doppler (RID) technique, whose essence is the parameters estimation, is developed to resolve it [3]. After three decades of research, there are mature processing methods for the motion compensation, such as the standard range alignment method for the translational range migration compensation, keystone transform for the migration through resolution cells compensation and phase gradient autofocus method for the phase focusing [5,6,7], while the scatterer-dependent Doppler spread compensation with the RID technique remains a great challenge [8,9,10]. This is because, in each range cell, multicomponent scatterers exist, and the RID technique (or the parameters estimation algorithm) needs overall consideration of the computational cost, cross term suppression, anti-noise performance, etc. [3,10,11,12,13,14,15,16]. Most researchers currently focus on the scatterer-dependent Doppler spread compensation with the RID technique [8,9,10,11,12], which is also the focus of this paper.

In general, after the motion compensation, azimuth echoes in a range cell of the ship target can be modeled as noisy multicomponent linear frequency-modulated (LFM) signals [8,9,10]. Nevertheless, in the harsh ocean environment, the LFM signal model is not suitable for ship targets. In references [12,13], simulations and analyses with the real radar data demonstrate that, for the ship targets in the harsh ocean environment, azimuth echoes in a range cell have to be modeled as noisy multicomponent cubic phase signals (CPSs). For ISAR imaging with the CPS model, the chirp rate and quadratic chirp rate induce the Doppler spread and defocus the ISAR image [14]. Therefore, now, the task of the RID technique is to estimate these two parameters and compensate the Doppler spread.

Until now, many researchers have studied the CPS and proposed several successful estimation algorithms. These estimation algorithms can be generally divided into three categories: linear algorithms [14], multilinear algorithms [12,13,17,18,19,20,21,22] and bilinear algorithms [23,24,25,26]. The linear algorithms, such as the modified discrete chirp Fourier transform for the CPS [14], employ three-dimensional brute-force searching to obtain a high anti-noise performance without the cross term, but this is at the cost of a high computational burden [10,12]. Multilinear algorithms employ the fourth-order autocorrelation function to reduce the phase order of the CPS, and then complete the two-dimensional energy accumulation with several operations, such as the fast Fourier transform (FFT), non-uniform FFT (NUFFT) [15,16] and decoupling techniques. Compared with linear algorithms, the multilinear algorithms have a lower computational cost [12]. This is the reason why many researchers currently focus on them. Representative multilinear algorithms include the product high-order ambiguity function [17], product generalized cubic phase function [18], integrated generalized cubic phase function (IGCPF) [19], scaled Fourier transform (SCFT)-based algorithm [13], noise-resistant parameter estimation algorithm [20], coherently IGCPF [21], generalized SCFT (GSCFT)-based algorithm [12] and generalized decoupling technique (GDT)-based algorithm [22]. However, the fourth-order autocorrelation function influences the cross term suppression and anti-noise performance seriously. Drawing lessons from LFM signal research, we know that the bilinear algorithm, which is based on the two-dimensionally coherent energy accumulation, can resolve the problems of the linear and multilinear algorithms [8,9,10]. Several bilinear algorithms have been developed for the CPS, such as the cubic phase function (CPF) [23,24], non-uniform sampled CPF [25] and local polynomial Wigner distribution [26]. Nevertheless, due to the quadratic chirp rate, these bilinear algorithms can only accumulate the energy along the delay axis and discard the energy along the slow time axis. Thus, spurious peaks can be easily induced and the anti-noise performance is poor. Several researchers have employed two-dimensional brute-force searching to improve these bilinear algorithms [27], although the two-dimensional brute-force searching makes the bilinear algorithm lose its inherent advantages [8]. In [28], we used the parameter space switching method to speed up the accumulation of the auto term. However, the accumulation is incoherent, and the resolution, cross term suppression and anti-noise performance are still poor.

In this paper, by employing the cubic phase bilinear autocorrelation function (CPBAF), NUFFT, operation of taking the complex modulus, inverse FFT (IFFT), GDT and FFT, a novel estimation algorithm, known as the integrated CPBAF (ICPBAF) is proposed for the CPS. The ICPBAF is bilinear and based on the two-dimensionally coherent energy accumulation. The bilinearity and two-dimensionally coherent accumulation of the ICPBAF guarantee the high cross term suppression and anti-noise performance. Compared to five other representative estimation algorithms including the CPF, IGCPF, SCFT-based algorithm, GSCFT-based algorithm and GDT-based algorithm, the ICPBAF can acquire higher cross term suppression and anti-noise performance with a moderate computational cost. Thereafter, with the ICPBAF, we present a RID ISAR imaging algorithm for the ship target. Through simulations and analyses with the synthetic model and the real radar data, we verify the effectiveness of the ICPBAF and corresponding RID ISAR imaging algorithm.

## 2. ISAR Imaging Geometry for the Ship Target

According to references [5,6,7,12,13], the ISAR imaging geometry described here is based on the assumption that the motion compensation has been implemented and only the scatterer-dependent Doppler spread is considered.

**Figure 1:**
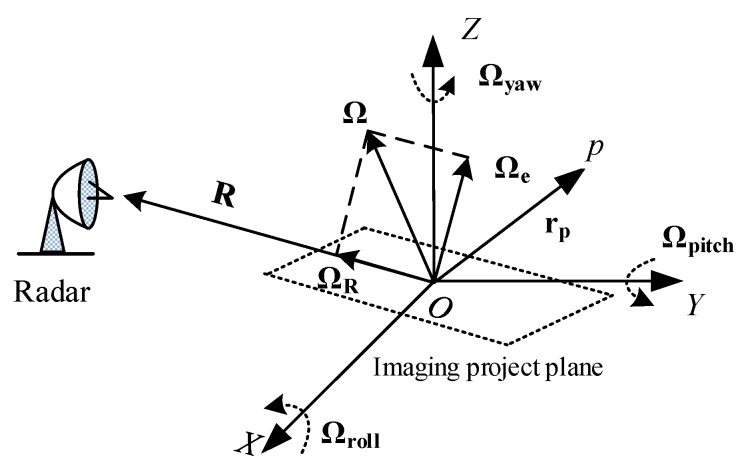
ISAR imaging geometry for the ship target.

Figure 1 shows the geometry for ISAR imaging of the ship target. The *X*, *Y* and *Z* axes of the Cartesian coordinate system overlap with the longitudinal, horizontal and vertical axes of the ship target, respectively. **R** is the unit vector of the radar line of sight (parallel to the range cell). $\Omega_{\mathrm{roll}}$, $\Omega_{\mathrm{pitch}}$ and $\Omega_{\mathrm{yaw}}$ denote the angular rotational vectors of the ship target rotating around the *X*, *Y* and *Z* axes, respectively. Ω is the synthetic vector of $\Omega_{\mathrm{roll}}$, $\Omega_{\mathrm{pitch}}$ and $\Omega_{\mathrm{yaw}}$, and can be decomposed into $\Omega_{e}$ and $\Omega_{R}$ in the plane determined by **R** and Ω. $\Omega_{R}$ does not cause the radial motion and thus has no effect on the phase of echoes, while $\Omega_{e}$ causes the change of the Doppler frequency and is called the effective rotational vector.

Considering the generic scatterer *p* with the directional vector $r_{p}$, we represent its Doppler frequency as:

(1) $$f_{D}=-\frac{2}{\lambda }\left[\left(r_{p}\times \Omega_{e}\right)•R\right]$$

where × and • represent the outer product and inner product, respectively. λ denotes the wavelength of the transmitted signal.

The effective rotational vector $\Omega_{e}$ is usually time-varying for the ship target. Based on the Weierstrass approximation principle [29] and analyses in [12], $\Omega_{e}$ of the ship target can be approximated as:

(2) $$\Omega_{e}\left(t_{m}\right)=\alpha +\beta t_{m}+\frac{1}{2}\gamma t_{m}^{2}$$

where $t_{m}$ is the slow time (or azimuth time). α, β and γ denote coefficients of the constant-, first-, and second-term of $\Omega_{e}$, respectively (or the angular velocity, acceleration and acceleration rate, respectively).

Substituting (2) into (1), we have:

(3) $$f_{D}=-\frac{2}{\lambda }\left\{\left(r_{p}\times \alpha \right)•R+\left[\left(r_{p}\times \beta \right)•R\right]t_{m}+\frac{1}{2}\left[\left(r_{p}\times \gamma \right)•R\right]t_{m}^{2}\right\}$$

With (3), after the motion compensation with the standard range alignment method, keystone transform and phase gradient autofocus method, the azimuth echo of the generic scatterer *p* [18,19] can be represented as:

(4) $$s_{p}\left(t_{m}\right)=\sigma_{p}\exp\left[j2\pi \left(\phi_{1,p}t_{m}+\frac{1}{2}\phi_{2,p}t_{m}^{2}+\frac{1}{6}\phi_{3,p}t_{m}^{3}\right)\right]$$

where $\sigma_{p}$ denotes the amplitude. $\phi_{1,p}=-2\left(r_{p}\times \alpha \right)•R/\lambda$, $\phi_{2,p}=-2\left(r_{p}\times \beta \right)•R/\lambda$ and $\phi_{3,p}=-2\left(r_{p}\times \gamma \right)•R/\lambda$ denote the centroid frequency, chirp rate and quadratic chirp rate, respectively.

The azimuth echo of the generic scatterer *p* takes the form of the CPS. In ISAR imaging, multicomponent scatterers coexist each range cell. Assuming the number of scatterers in the *i*-th (1≤i≤I) range cell is P and taking the additive complex white Gaussian noise $n_{i}\left(t_{m}\right)$ into account, we can represent azimuth echoes in the *i*-th range cell as noisy multicomponent CPSs:

(5) $$s_{i}\left(t_{m}\right)=\sum_{p=1}^{P}s_{p}\left(t_{m}\right)+n_{i}\left(t_{m}\right)=\sum_{p=1}^{P}\sigma_{p}\exp\left[j2\pi \left(\phi_{1,p}t_{m}+\frac{1}{2}\phi_{2,p}t_{m}^{2}+\frac{1}{6}\phi_{3,p}t_{m}^{3}\right)\right]+n_{i}\left(t_{m}\right)$$

Obviously, scatterers at different coordinates correspond to different centroid frequencies in each range cell and we can use this characteristic to construct ISAR image of the ship target [3]. Nevertheless, the chirp rate and quadratic chirp rate also exist in (5) and will induce the Doppler spread (degrade the cross-range resolution). Thus, now, the task is to estimate these two parameters and compensate the corresponding Doppler spread.

## 3. ICPBAF for Multicomponent CPSs

In order to construct a well-focused ISAR image of the ship target, the ICPBAF is proposed for the parameters estimation of the CPS. The principle of the ICPBAF and its cross term characteristic are discussed in this section.

### 3.1. Principle of the ICPBAF

According to the reference [24], the CPBAF of (5) is presented as:

(6) $$\begin{array}{ll} R_{i}\left(t_{m},\tau_{m}\right) & =s_{i}\left(t_{m}-\tau_{m}\right)s_{i}\left(t_{m}+\tau_{m}\right)\\ & =\underset{\text{the auto tern}}{\underset{︸}{\sum_{p=1}^{P}\sigma_{p}^{2}\exp\left[j2\pi \left(2\phi_{1,p}t_{m}+\phi_{2,p}t_{m}^{2}+\frac{1}{3}\phi_{3,p}t_{m}^{3}\right)\right]\exp\left[j2\pi \left(\phi_{2,p}+\phi_{3,p}t_{m}\right)\tau_{m}^{2}\right]}}+R_{i,cros}\left(t_{m},\tau_{m}\right)+n_{R,i}\left(t_{m},\tau_{m}\right) \end{array}$$

where $\tau_{m}$ denotes the lag variable. $R_{i,cros}\left(t_{m},\tau_{m}\right)$ and $n_{R,i}\left(t_{m},\tau_{m}\right)$ denote the cross term and noise, respectively.

In the auto term (corresponding to the CPS) of the CPBAF, the slow time $t_{m}$ and lag $\tau_{m}$ nonlinearly couple with each other. If the first exponent of the auto term does not exist, we can employ the decoupling technique GDT to eliminate the coupling and then, accumulate the signal energy coherently with the Fourier transform along the $\tau_{m}$ axis. That is, the first exponent of the auto term disables the GDT and the following Fourier transform-based coherent accumulation. It is easily seen from (6) that, due to the coupling, the Fourier transform along the $\tau_{m}$ axis can accumulate the signal energy into the inclined line, which benefits the signal detection and parameters estimation [28]. Here, we employ this characteristic and perform the Fourier transform along the $\tau_{m}$ axis. However, the $\tau_{m}$ axis corresponds to the nonuniform $\tau_{m}^{2}$ and the FFT is no longer applicable. Fortunately, we can adopt the NUFFT to speed up the Fourier transform of the nonuniform data without the performance loss. Readers can refer to references [15,16] for more details about the NUFFT:

(7) $$\begin{array}{ll} G_{i}\left(t_{m},f_{\tau_{m}^{2}}\right) & ={\mathrm{NUFFT}}_{\tau_{m}^{2}}\left[R_{i}\left(t_{m},\tau_{m}\right)\right]\\ & =\underset{\text{the auto tern}}{\underset{︸}{\sum_{p=1}^{P}\sigma_{p}^{2}\exp\left[j2\pi \left(2\phi_{1,p}t_{m}+\phi_{2,p}t_{m}^{2}+\frac{1}{3}\phi_{3,p}t_{m}^{3}\right)\right]\delta \left(f_{\tau_{m}^{2}}-\phi_{2,p}-\phi_{3,p}t_{m}\right)}}+G_{i,cros}\left(t_{m},f_{\tau_{m}^{2}}\right)+n_{G,i}\left(t_{m},f_{\tau_{m}^{2}}\right) \end{array}$$

where $f_{\tau_{m}^{2}}$ is the frequency domain with respect to $\tau_{m}$. δ(•) is the Dirac delta function. $G_{i,cros}\left(t_{m},f_{\tau_{m}^{2}}\right)$ and $n_{G,i}\left(t_{m},f_{\tau_{m}^{2}}\right)$ denote the cross tern and noise after the NUFFT, respectively.

In (7), the energy of the auto term peaks along the inclined line $f_{\tau_{m}^{2}}-\phi_{2,p}-\phi_{3,p}t_{m}=0$. In general, we can accumulate the signal energy along this line by employing the Radon transform or Hough transform. However, this kind of processing methods is incoherent and the two-dimensionally brute-force searching will influence the efficiency [8,10,12]. In order to accumulate the signal energy coherently, we take the complex modulus of $G_{i}\left(t_{m},f_{\tau_{m}^{2}}\right)$ and perform the IFFT with respect to $f_{\tau_{m}^{2}}$:

(8) $$\begin{array}{ll} Q_{i}\left(t_{m},b_{m}\right) & ={\mathrm{IFFT}}_{f_{\tau_{m}^{2}}}\left[\left|G_{i}\left(t_{m},f_{\tau_{m}^{2}}\right)\right|\right]\\ & \approx \underset{\text{the auto term}}{\underset{︸}{\sum_{p=1}^{P}\sigma_{p}^{2}\exp\left[j2\pi \left(\phi_{2,p}+\phi_{3,p}t_{m}\right)b_{m}\right]}}+Q_{i,cros}\left(t_{m},b_{m}\right)+n_{Q,i}\left(t_{m},b_{m}\right) \end{array}$$

where $b_{m}$ is the time domain with respect to $f_{\tau_{m}^{2}}$.|•| denotes the operation of taking the complex modulus. $Q_{i,cros}\left(t_{m},b_{m}\right)$ and $n_{Q,i}\left(t_{m},b_{m}\right)$ denote the cross term and noise, respectively. In realistic applications, the signal length is finite and the sinc function should be used instead of the Dirac function. Under this condition, taking the modulus bends the negative side lobes to the positive and taking the IFFT may induce a different equation than (8). However, the energy of the sinc function concentrates in the main lobe and the effectiveness of negative side lobes is very small. Therefore, to be exact, we use the approximation ≈ in (8).

In (8), the operation of taking the complex modulus eliminates the disturbance $\exp\left[j2\pi \left(2\phi_{1,p}t_{m}+\phi_{2,p}t_{m}^{2}+\left(\phi_{3,p}/3\right)t_{m}^{3}\right)\right]$ and the IFFT transforms $\left|G_{i}\left(t_{m},f_{\tau_{m}^{2}}\right)\right|$ back into the form of the exponent. Obviously, we can employ the GDT to eliminate the linear coupling between $t_{m}$ and $b_{m}$ in the auto term of $Q_{i}\left(t_{m},b_{m}\right)$. According to the reference [22], the GDT takes the form:

(9) $$\begin{array}{cc} D\left(f_{\left[g\left(\Delta_{m}\right){ϒ}_{m}^{b}\right]},\Delta_{m}\right) & =\int_{{ϒ}_{m}}\left\{u\left(\Delta_{m}\right)\exp\left[j2\pi \phi g\left(\Delta_{m}\right){ϒ}_{m}^{b}\right]\right\}\times \exp\left[-j2\pi \xi g\left(\Delta_{m}\right){ϒ}_{m}^{b}f_{\left[g\left(\Delta_{m}\right){ϒ}_{m}^{b}\right]}\right]d\left[{ϒ}_{m}\right]\\ & =u\left(\Delta_{m}\right)\delta \left(f_{\left[g\left(\Delta_{m}\right){ϒ}_{m}^{b}\right]}-\frac{\phi }{\xi }\right) \end{array}$$

where $g\left(\Delta_{m}\right)$ and $u\left(\Delta_{m}\right)$ are both functions of the variable $\Delta_{m}$. $f_{\left[g\left(\Delta_{m}\right){ϒ}_{m}^{b}\right]}$ is the scaled frequency domain with respect to ${ϒ}_{m}$. b is a known constant. ξ denotes the zoom factor. ϕ is an unknown parameter.

Based on the coupling in (8) and the GDT in (9), several substitutions are done as follows:

(10) $$\{\begin{array}{c} g\left(\Delta_{m}\right)=b_{m},{ϒ}_{m}=t_{m},b=1,\xi =1\\ u\left(\Delta_{m}\right)\exp\left[j2\pi \phi g\left(\Delta_{m}\right){ϒ}_{m}^{b}\right]=Q_{i}\left(t_{m},b_{m}\right) \end{array}$$

With substitutions in (10), we apply the GDT to $Q_{i}\left(t_{m},b_{m}\right)$:

(11) $$\begin{array}{ll} \Gamma_{i}\left(f_{\left[b_{m}t_{m}\right]},b_{m}\right) & =\int_{t_{m}}Q_{i}\left(t_{m},b_{m}\right)\exp\left[-j2\pi f_{\left[b_{m}t_{m}\right]}b_{m}t_{m}\right]d\left(t_{m}\right)\\ & =\underset{\text{the auto tern}}{\underset{︸}{\sum_{p=1}^{P}\sigma_{p}^{2}\exp\left(j2\pi \phi_{2,p}b_{m}\right)\delta \left(f_{\left[b_{m}t_{m}\right]}-\phi_{3,p}\right)}}+\Gamma_{i,cros}\left(f_{\left[b_{m}t_{m}\right]},b_{m}\right)+n_{\Gamma ,i}\left(f_{\left[b_{m}t_{m}\right]},b_{m}\right) \end{array}$$

where $f_{\left[b_{m}t_{m}\right]}$ is the scaled frequency domain with respect to $t_{m}$. $\Gamma_{i,cros}\left(f_{\left[b_{m}t_{m}\right]},b_{m}\right)$ and $n_{\Gamma ,i}\left(f_{\left[b_{m}t_{m}\right]},b_{m}\right)$ denote the cross term and noise after the GDT, respectively.

The GDT in (11) can be implemented by using the IFFT-based CZT. More details about the fast implementation of the GDT can be found in the reference [22]. After the GDT, the energy of the auto term is accumulated into the beeline $f_{\left[b_{m}t_{m}\right]}-\phi_{3,p}=0$. Now, we perform the FFT along the $b_{m}$ axis and obtain the ICPBAF:

(12) $$\begin{array}{ll} \Psi_{i}\left(f_{\left[b_{m}t_{m}\right]},f_{b_{m}}\right) & ={\mathrm{FFT}}_{b_{m}}\left[\Gamma_{i}\left(f_{\left[b_{m}t_{m}\right]},b_{m}\right)\right]\\ & =\underset{\text{the auto tern}}{\underset{︸}{\sum_{p=1}^{P}\sigma_{p}^{2}\delta \left(f_{b_{m}}-\phi_{2,p}\right)\delta \left(f_{\left[b_{m}t_{m}\right]}-\phi_{3,p}\right)}}+\Psi_{i,cros}\left(f_{\left[b_{m}t_{m}\right]},f_{b_{m}}\right)+n_{\Psi ,i}\left(f_{\left[b_{m}t_{m}\right]},f_{b_{m}}\right) \end{array}$$

where $f_{b_{m}}$ is the frequency domain with respect to $b_{m}$. $\Psi_{i,cros}\left(f_{\left[b_{m}t_{m}\right]},f_{b_{m}}\right)$ and $n_{\Psi ,i}\left(f_{\left[b_{m}t_{m}\right]},f_{b_{m}}\right)$ denote the cross term and noise after the FFT, respectively.

For the ICPBAF, the auto term peaks at ($\phi_{2,p}$,$\phi_{3,p}$) on the chirp rate-quadratic chirp rate domain. Parameters $\phi_{2,p}$ and $\phi_{3,p}$ can be estimated by constructing a cost function to (12) [23]. With these two estimated parameters, other parameters ($\sigma_{p}$ and $\phi_{1,p}$) can be obtained by the dechirping and FFT operations [23].

According to (6)–(8), (11) and (12), we give the abbreviated expression of the ICPBAF:

(13) $$\Psi_{i}\left(f_{\left[b_{m}t_{m}\right]},f_{b_{m}}\right)={\mathrm{FFT}}_{b_{m}}\left\{{\mathrm{GDT}}_{b_{m}t_{m}}\left\{{\mathrm{IFFT}}_{f_{\tau_{m}^{2}}}\left\{\left|{\mathrm{NUFFT}}_{\tau_{m}^{2}}\left[\mathrm{CPBAF}\left[s_{i}\left(t_{m}\right)\right]\right]\right|\right\}\right\}\right\}$$

Analyses above indicate that the ICPBAF is a coherent bilinear algorithm and can be implemented by using the complex multiplication, FFT, IFFT and NUFFT. The two-dimensionally coherent accumulation and bilinearity guarantee the low computational cost, high anti-noise performance and cross term suppression of the ICPBAF, which will be demonstrated in Section 4. The cross term characteristic analysis can demonstrate whether the cross term can accumulate as the auto term or not [10,12]. Further, with the cross term characteristic analysis, we can also have a more in-depth understanding of the cross term suppression. Therefore, we analyze the cross term characteristic of the ICPBAF in the following section.

### 3.2. Cross Term Characteristic Analysis

In order to formulate the cross term problem arising from multicomponent CPSs, we consider the noise-free signal with two components, l∈[1,P−1] and q∈[l+1,P], which is denoted as:

(14) $$s_{l,q}\left(t_{m}\right)=\sigma_{l}\exp\left[j2\pi \left(\phi_{1,l}t_{m}+\frac{1}{2}\phi_{2,l}t_{m}^{2}+\frac{1}{6}\phi_{3,l}t_{m}^{3}\right)\right]+\sigma_{q}\exp\left[j2\pi \left(\phi_{1,q}t_{m}+\frac{1}{2}\phi_{2,q}t_{m}^{2}+\frac{1}{6}\phi_{3,q}t_{m}^{3}\right)\right]$$

The CPBAF of $s_{l,q}\left(t_{m}\right)$ can be presented as:

(15) $$R_{l,q}\left(t_{m},\tau_{m}\right)=R_{l,q,auto}\left(t_{m},\tau_{m}\right)+R_{l,q,cros}\left(t_{m},\tau_{m}\right)$$

where:

(15a) $$\begin{array}{cc} R_{l,q,auto}\left(t_{m},\tau_{m}\right) & =\sigma_{l}^{2}\exp\left[j2\pi \left(2\phi_{1,l}t_{m}+\phi_{2,l}t_{m}^{2}+\frac{1}{3}\phi_{3,l}t_{m}^{3}\right)\right]\exp\left[j2\pi \left(\phi_{2,l}+\phi_{3,l}t_{m}\right)\tau_{m}^{2}\right]\\ & +\sigma_{q}^{2}\exp\left[j2\pi \left(2\phi_{1,q}t_{m}+\phi_{2,q}t_{m}^{2}+\frac{1}{3}\phi_{3,q}t_{m}^{3}\right)\right]\exp\left[j2\pi \left(\phi_{2,q}+\phi_{3,q}t_{m}\right)\tau_{m}^{2}\right] \end{array}$$

(15b) $$\begin{array}{cc} R_{l,q,cros}\left(t_{m},\tau_{m}\right) & =2\sigma_{l}\sigma_{q}\exp\left\{j2\pi \left[\left(\phi_{1,l}+\phi_{1,q}\right)t_{m}+\frac{1}{2}\left(\phi_{2,l}+\phi_{2,q}\right)t_{m}^{2}+\frac{1}{6}\left(\phi_{3,l}+\phi_{3,q}\right)t_{m}^{3}\right]\right\}\cos\{2\pi [(\phi_{1,l}-\phi_{1,q}+\left(\phi_{2,l}-\phi_{2,q}\right)t_{m}\\ & +\frac{1}{2}\left(\phi_{3,l}-\phi_{3,q}\right)t_{m}^{2})\tau_{m}+\frac{1}{6}\left(\phi_{3,l}-\phi_{3,q}\right)\tau_{m}^{3}]\}\exp\left\{j2\pi \left[\frac{1}{2}\left(\phi_{2,l}+\phi_{2,q}\right)+\frac{1}{2}\left(\phi_{3,l}+\phi_{3,q}\right)t_{m}\right]\tau_{m}^{2}\right\} \end{array}$$

Performing the NUFFT, operation of taking the complex modulus, IFFT, GDT and FFT on $R_{l,q}\left(t_{m},\tau_{m}\right)$, we have:

(16) $$\Psi_{l,q}\left(f_{\left[b_{m}t_{m}\right]},f_{b_{m}}\right)={\mathrm{FFT}}_{b_{m}}\left\{{\mathrm{GDT}}_{b_{m}t_{m}}\left\{{\mathrm{IFFT}}_{f_{\tau_{m}^{2}}}\left\{\left|{\mathrm{NUFFT}}_{\tau_{m}^{2}}\left[R_{l,q,auto}\left(t_{m},\tau_{m}\right)+R_{l,q,cros}\left(t_{m},\tau_{m}\right)\right]\right|\right\}\right\}\right\}$$

With careful analyses of Equations (15a) and (15b), we find that, compared to the auto term $R_{l,q,auto}\left(t_{m},\tau_{m}\right)$, the cosine function of $R_{l,q,cros}\left(t_{m},\tau_{m}\right)$ will disturb the NUFFT, operation of taking the complex modulus, IFFT, GDT and FFT. According to the characteristic of the cosine function, when the Equation (17) is satisfied for every $t_{m}$ and $\tau_{m}$, the influence of the cosine function will be eliminated:

(17) $$\left(\phi_{1,l}-\phi_{1,q}+\left(\phi_{2,l}-\phi_{2,q}\right)t_{m}+\frac{1}{2}\left(\phi_{3,l}-\phi_{3,q}\right)t_{m}^{2}\right)\tau_{m}+\frac{1}{6}\left(\phi_{3,l}-\phi_{3,q}\right)\tau_{m}^{3}=n\pi ,n=\cdots -2,-1,0,1,2,\cdots$$

Obviously, only when $\phi_{1,l}=\phi_{1,q}$, $\phi_{2,l}=\phi_{2,q}$ and $\phi_{3,l}=\phi_{3,q}$, Equation (17) equals to zero for every $t_{m}$ and $\tau_{m}$. That is, different from the auto term, the cross term of the ICPBAF cannot be accumulated. Thus, when the signal length is infinite, compared to the auto term, the cross term can be ignored, i.e., the ICPBAF in (12) can be approximated as:

(18) $$\Psi_{i}\left(f_{\left[b_{m}t_{m}\right]},f_{b_{m}}\right)=\underset{\text{the auto tern}}{\underset{︸}{\sum_{p=1}^{P}\sigma_{p}^{2}\delta \left(f_{b_{m}}-\phi_{2,p}\right)\delta \left(f_{\left[b_{m}t_{m}\right]}-\phi_{3,p}\right)}}+n_{\Psi ,i}\left(f_{\left[b_{m}t_{m}\right]},f_{b_{m}}\right)$$

This section gives the basic analyses of the ICPBAF, including the principle of the ICPBAF and its cross term characteristic analysis. In order to illustrate how the ICPBAF works under multicomponent CPSs, we give a numerical example in the following. Considering realistic applications, this numerical example includes two situations, multicomponent CPSs with the same amplitude and multicomponent CPSs with the different amplitudes.

**Figure 2:**
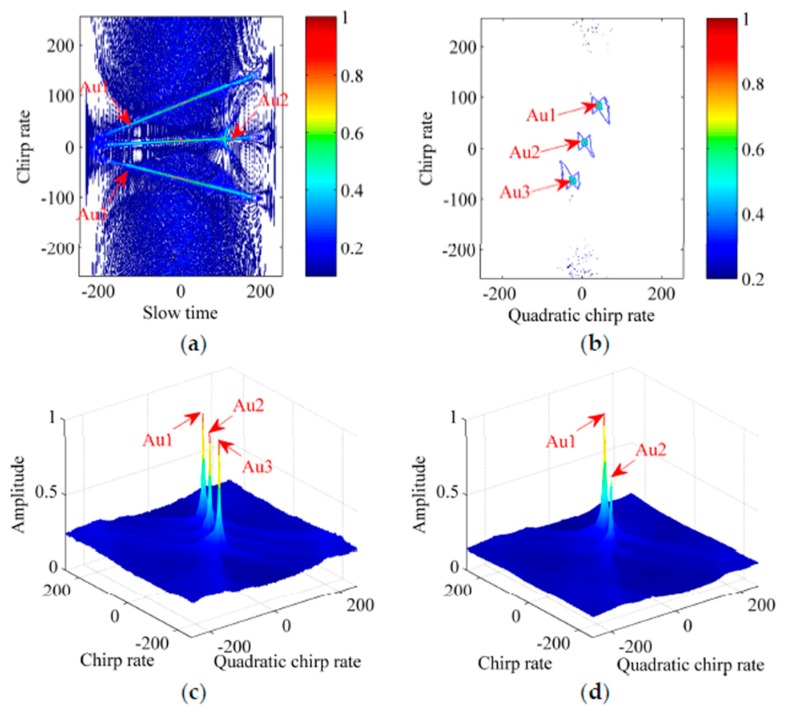
Simulation results of Example 1. (**a**) Contour of the slow time-chirp rate distribution; (**b**) Contour of the ICPBAF; (**c**) Stereogram of the ICPBAF; (**d**) Stereogram of the ICPBAF under *Au1*, *Au2* and *Au3* with the different amplitudes.

**Example** **1.** *We consider three noise-free CPSs denoted by Au1, Au2 and Au3. The sampling frequency* $F_{t_{m}}$ *[same as the pulse repetition frequency (PRF)] is 256 Hz, and the signal length* $N_{t_{m}}$ *(same as the echo pulses) is equal to 512. The signal parameters are set as follows:* $\phi_{1,1}$ *= 100 Hz,* $\phi_{2,1}$ *= 84 Hz/s,* $\phi_{3,1}$ *= 80 Hz/s^2^ for Au1;* $\phi_{1,2}$ *= 20 Hz,* $\phi_{2,2}$ *= 12 Hz/s,* $\phi_{3,2}$ *= 10 Hz/s^2^ for Au2, and* $\phi_{1,3}$ *= −80 Hz,* $\phi_{2,3}$ *= −64 Hz/s,* $\phi_{3,3}$ *= −50 Hz/s^2^ for Au3. Results of Au1, Au2 and Au3 with the same amplitude (*$\sigma_{1}=\sigma_{2}=\sigma_{3}=1$*) are provided in Figure 2a–c, while Figure 2d shows the ICPBAF under Au1, Au2 and Au3 with the different amplitudes (*$\sigma_{1}=1$*,* $\sigma_{2}=0.8$*,* $\sigma_{3}=0.1$*). It is known that actual values of parameters are related with the position of the peak in the figure, sampling frequency and signal length. Thus, values in figures are relative and units of measurements are not added.*

By performing the NUFFT along the $\tau_{m}$ axis of the CPBAF in (6), we obtain the slow time-chirp rate distribution in Figure 2a. The bilinearity of the CPBAF causes the situation that the auto term and cross term coexist. In Figure 2a, we can find that, due to the coupling between $t_{m}$ and $\tau_{m}$, the auto term takes the form of three inclined lines. In order to accumulate the auto term, we take the complex modulus, and then, perform the IFFT, GDT and FFT sequentially. Figure 2b shows the contour of the ICPBAF and its stereogram is shown in Figure 2c. Obviously, as analyzed above, the auto term accumulates into the ideal spread function, while the cross term cannot accumulate and even can be ignored. In Figure 2c, with the peak detection technique [22,23], ($\phi_{2,1}$, $\phi_{3,1}$), ($\phi_{2,2}$, $\phi_{3,2}$) and ($\phi_{2,3}$, $\phi_{3,3}$) are estimated as (84 Hz/s, 80 Hz/s^2^), (12 Hz/s, 10 Hz/s^2^) and (−64 Hz/s, −50 Hz/s^2^), respectively. Thereafter, compensating the phase term pertaining to the estimated parameters and performing the FFT, we estimate ($\sigma_{1}$, $\phi_{1,1}$), ($\sigma_{2}$, $\phi_{1,2}$) and ($\sigma_{3}$, $\phi_{1,3}$) as (1, 100 Hz), (1, 20 Hz) and (1, −80 Hz), respectively.

Figure 2a–c consider multicomponent CPSs with the same amplitude. However, the amplitudes are always different in realistic applications. Figure 2d considers multicomponent CPSs with different amplitudes. With the result shown in Figure 2d, we know that, when the differences between amplitudes are large, the auto terms of weak CPSs may be submerged in the residual cross terms generated by the strong CPSs and the Clean technique [10,12,13] should be employed to separate the strong and weak CPSs.

## 4. Performance Analysis of the ICPBAF

The computational cost, cross term suppression and anti-noise performance play important roles in the parameters estimation algorithm [22,23,24]. In this section, we analyze the ICPBAF from these three aspects, and some comparisons with other five representative algorithms including the CPF, IGCPF, SCFT-based algorithm, GSCFT-based algorithm and GDT-based algorithm will be performed.

### 4.1. Computational Cost Analysis

Assume the length of slow time samples $N_{t_{m}}$ is equal to the length of lag samples $N_{\tau_{m}}$. The ICPBAF implementation needs the CPBAF [$O\left(N_{t_{m}}^{2}\right)$], NUFFT along $\tau_{m}$[$O\left(N_{t_{m}}^{2}\log_{2}N_{t_{m}}\right)$], operation of taking the complex modulus [$O\left(N_{t_{m}}^{2}\right)$], IFFT along $f_{\tau_{m}^{2}}$[$O\left(N_{t_{m}}^{2}\log_{2}N_{t_{m}}\right)$], GDT [$O\left(N_{t_{m}}^{2}\log_{2}N_{t_{m}}\right)$] and FFT along $b_{m}$[$O\left(N_{t_{m}}^{2}\log_{2}N_{t_{m}}\right)$]. Therefore, the overall computational cost of the ICPBAF is in the order of $O\left(N_{t_{m}}^{2}\log_{2}N_{t_{m}}\right)$ and listed in Table 1, which also gives computational costs of five other representative algorithms, the CPF, IGCPF, SCFT-based algorithm, GSCFT-based algorithm and GDT-based algorithm.

Referring to references [22,23], we know that the CPF only uses the discrete Fourier transform to accumulate the CPBAF along $\tau_{m}$ and discards the energy along $t_{m}$. Compared to the ICPBAF, its low computational cost is at the cost of the low cross term suppression and anti-noise performance. The IGCPF and SCFT-based algorithm need higher computational costs than the GSCFT-based algorithm, GDT-based algorithm and ICPBAF. This is because the Fourier transform along the non-uniform $\tau_{m}$ axis of these two algorithms are not speed up [13,19]. The GSCFT-based algorithm, GDT-based algorithm and ICPBAF need the similar computational cost. In the following two sections, we will demonstrate that the ICPBAF has superiorities in the cross term suppression and anti-noise performance. We can refer to references [12,13,19,22,23,24] for more details about these five referenced algorithms.

### 4.2. Cross Term Suppression Analysis

Analyses in Section 3.2 demonstrate that the cross term cannot accumulate as the auto term. In this section, we uses the numerical example below to demonstrate the high cross term suppression of the ICPBAF.

**Figure 3:**
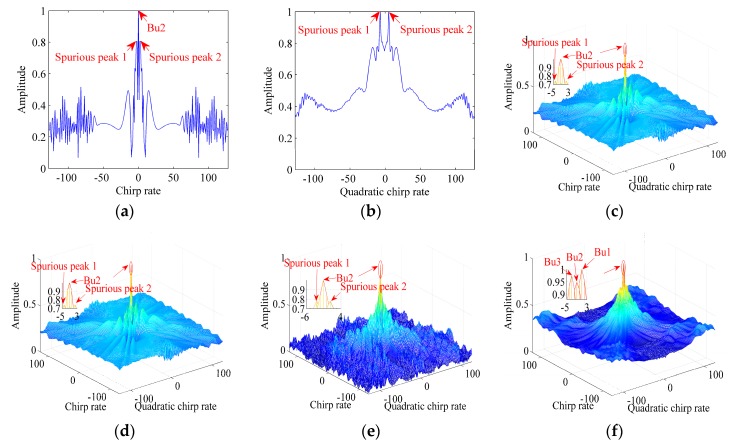
Simulation results of Example 2. (**a**) Simulation result of the CPF; (**b**) Simulation result of the IGCPF; (**c**) Simulation result of the SCFT-based algorithm; (**d**) Simulation result of the GSCFT-based algorithm; (**e**) Simulation result of the GDT-based algorithm; (**f**) Simulation result of the ICPBAF.

**Example** **2.** *We consider three noise-free CPSs denoted by Bu1, Bu2 and Bu3.* $F_{t_{m}}$ *and* $N_{t_{m}}$ *are 128 Hz and 256, respectively. The signal parameters are set as follows:* $\sigma_{1}=1$*,* $\phi_{1,1}$ *= 10 Hz,* $\phi_{2,1}$ *= 2 Hz/s,* $\phi_{3,1}$ *= 2 Hz/s^2^ for Bu1;* $\sigma_{2}=1$*,* $\phi_{1,2}$ *= 6 Hz,* $\phi_{2,2}$ *= 0 Hz/s,* $\phi_{3,2}$ *= −2 Hz/s^2^ for Bu2, and* $\sigma_{3}=1$*,* $\phi_{1,3}$ *= 2 Hz,* $\phi_{2,3}$ *= −2 Hz/s,* $\phi_{3,3}$ *= −6 Hz/s^2^ for Bu3. Figure 3 gives simulation results of the CPF, IGCPF, SCFT-based algorithm, GSCFT-based algorithm, GDT-based algorithm and ICPBAF.*

Obviously, in Figure 3, only the ICPBAF can obtain correct positions of *Bu1*, *Bu2* and *Bu3*. The CPF is bilinear, while it discards the energy along $t_{m}$. Although the IGCPF, SCFT-based algorithm, GSCFT-based algorithm and GDT-based algorithm employ the two-dimensional energy accumulation to suppress the cross term, their fourth-order autocorrelation functions influence the cross term suppression seriously. In this example, their fourth-order autocorrelation functions induce the cross term with 78 components. Note that, under some special situations, the cross terms of the CPF and IGCPF can accumulate as their auto terms [19,30]. Compared to the CPF, IGCPF, SCFT-based algorithm, GSCFT-based algorithm and GDT-based algorithm, the ICPBAF is bilinear and employs the two-dimensionally coherent energy accumulation. Therefore, as shown in Figure 3, the ICPBAF acquires the highest cross term suppression.

### 4.3. Anti-Noise Performance Analysis

The ICPBAF inevitably suffers from the estimation error under the presence of the noise. In this section, combing with a numerical example, we will demonstrate the high anti-noise performance of the ICPBAF. The input-output signal-to-noise ratio (SNR) ($SNR_{out}$ is listed in (19)) [31] and mean square error (MSE) [32,33] are utilized as measures of the noise resistance:

(19) $${\mathrm{SNR}}_{out}=10\log_{10}\frac{\sigma_{p}^{2}}{N_{t_{m}}\sigma^{2}}{\left\{{\left|\sum_{m=-\frac{N_{t_{m}}}{2}}^{\frac{N_{t_{m}}}{2}-1}s_{i}\left(m\right)\exp\left[-j\pi a_{2.p}^{\prime }{\left(\frac{m}{F_{t_{m}}}\right)}^{2}-j\pi \frac{a_{3,p}^{\prime }}{3}{\left(\frac{m}{F_{t_{m}}}\right)}^{3}\right]\right|}_{\max}\right\}}^{2}$$

where $\sigma^{2}$ is the power of the complex white Gaussian noise. $a_{2.p}^{\prime }$ and $a_{3,p}^{\prime }$ are estimations.

**Figure 4:**
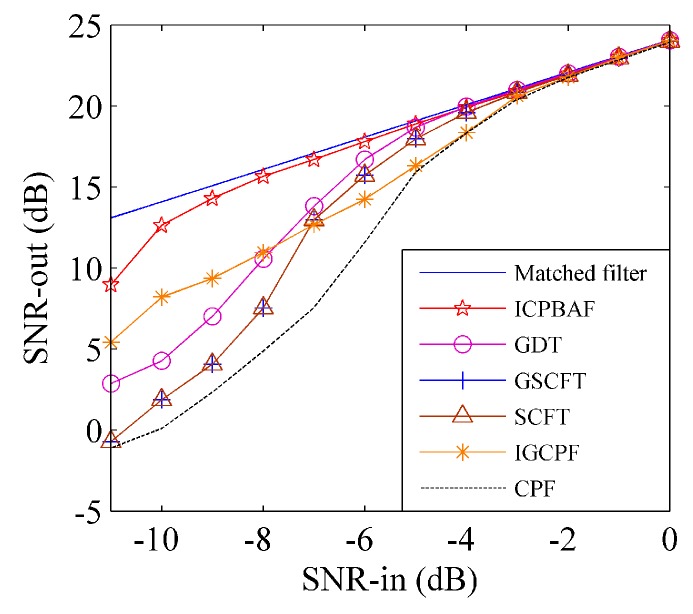
Input-output SNR comparison.

**Figure 5:**
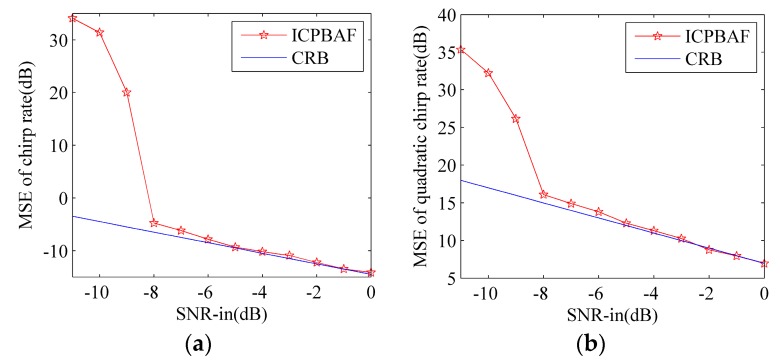
MSEs of the chirp rate and quadratic chirp rate estimations. (**a**) MSE of the chirp rate estimation; (**b**) MSE of the quadratic chirp rate estimation.

**Example** **3.** *We consider a CPS denoted by Cu.* $F_{t_{m}}$ *and* $N_{t_{m}}$ *are 256 Hz and 256, respectively. The signal parameters are set as follows:* $\phi_{1,1}$ *= 106 Hz,* $\phi_{2,1}$ *= 100 Hz/s,* $\phi_{3,1}$ *= 80 Hz/s^2^ for Cu. The tested input SNRs are SNR_in_ = [−11:1:0] and 200 trials have been performed for each SNR_in_ under the ten-time interpolation. Figure 4 and Figure 5 gives the input-output SNR and MSE, respectively.*

In Figure 4, threshold SNRs of the ICPBAF, GDT-based algorithm, GSCFT-based algorithm, SCFT-based algorithm, IGCPF and CPF are −8 dB, −5 dB, −3 dB, −3 dB, −2 dB and −2 dB, respectively. The bilinear CPF discards the energy accumulation along $t_{m}$ and references [23,24] has simulated its threshold SNR. The IGCPF employs the two-dimensional energy accumulation, while the accumulation is incoherent and its autocorrelation function is fourth-order [19]. Thus, its threshold SNR is also −2 dB and no better than that of the CPF. Note that, the CPF and IGCPF estimate the chirp rate and quadratic chirp rate separately, and thus the propagation of the estimation error exists in these two algorithms. Although the GSCFT-based algorithm, SCFT-based algorithm and GDT-based algorithm employ the two-dimensionally coherent energy accumulation and do not encounter the estimation error propagation, their fourth-order autocorrelation functions influence the anti-noise performance seriously. In Figure 4, the superiority of the ICPBAF in the anti-noise performance is obvious. This is because (1) the ICPBAF is bilinear; (2) the estimation error propagation does not exist, and (3) the two-dimensional energy accumulation is coherent. Above analyses also apply to noisy multicomponent CPSs. We consider noisy CPSs with two components. The GDT-based algorithm, GSCFT-based algorithm, SCFT-based algorithm and IGCPF are based on fourth-order autocorrelation functions, and the number of noise items is 65. However, the ICPBAF is bilinear and the number of noise items is 5. Although the CPF is bilinear and the number of noise items is 5, CPF is based on one-dimensional energy accumulation.

As functions of the input SNR, the observed MSEs for the chirp rate and quadratic chirp rate estimations are plotted in Figure 5a,b, respectively. The corresponding Cramer-Rao bounds (CRBs) are also shown in solid lines and their expressions can be found in [24,31,32].

As expected, the observed MSEs of the chirp rate and quadratic chirp rate estimations are inversely proportional to the input SNRs in Figure 5. MSEs of the chirp rate and the quadratic chirp rate estimations are close to CRB when SNR ≥ −8 dB. Results shown in Figure 5 verify the high anti-noise performance of the ICPBAF and also validate the result shown in Figure 4. 

## 5. RID ISAR Imaging Algorithm Based on the ICPBAF

Analyses and simulations in Section 4 demonstrate that, compared to the CPF, IGCPF, SCFT-based algorithm, GSCFT-based algorithm and GDT-based algorithm, the ICPBAF is more suitable for the parameters estimation of noisy multicomponent CPSs. In this section, based on the ICPBAF, a RID ISAR imaging algorithm is presented for the ship target. Detailed implementation procedures are given as follows.

*Step 1*: Complete the range compression for radar echoes with the matched filter $H\left(\hat{t}\right)=\mathrm{rect}\left(\hat{t}/T_{s}\right)\exp\left(j\pi \gamma {\hat{t}}^{2}\right)$ (where $\hat{t}$, $T_{s}$ and γ denote the fast time, pulse width and frequency modulation rate, respectively).
*Step 2*: Employ the standard range alignment method [5], keystone transform [6] and phase gradient autofocus method [7] to complete the motion compensation.
*Step 3*: Extract the data $s_{i}\left(t_{m}\right)$ in the *i*-th range cell.
*Step 4*: Calculate the energy of the extracted data $s_{i}\left(t_{m}\right)$. If the energy is smaller than the set threshold $E_{s}$ [8,12], set i=i+1 and repeat Step 3 until i=I.
*Step 5*: Apply the ICPBAF to $s_{i}\left(t_{m}\right)$ and estimate $\phi_{2,p}$ and $\phi_{3,p}$ with the peak detection technique [23,24].
*Step 6*: Dechirp $s_{i}\left(t_{m}\right)$ with $\exp\left[-j2\pi \left(\frac{\phi_{2,p}^{\prime }}{2}t_{m}^{2}+\frac{\phi_{3,p}^{\prime }}{6}t_{m}^{3}\right)\right]$, and then estimate $\phi_{1,p}$ and $\sigma_{p}$ via the FFT and peak detection technique.
  (20) $$\left(\sigma_{p}^{\prime }=\frac{A^{\prime }}{N_{t_{m}}},\phi_{1,p}^{\prime }=f_{t_{m}}^{\prime }\right)=\begin{array}{c} \arg\max\\ \left(A,f_{t_{m}}\right) \end{array}\left|{\mathrm{FFT}}_{t_{m}}\left(s_{i}\left(t_{m}\right)\exp\left[j2\pi \left(-\frac{\phi_{2,p}^{\prime }}{2}t_{m}^{2}-\frac{\phi_{3,p}^{\prime }}{6}t_{m}^{3}\right)\right]\right)\right|$$
  where $\sigma_{p}^{\prime }$ and $\phi_{1,p}^{\prime }$ denote estimations of the centroid frequency and amplitude for the *p*th CPS, respectively. $A^{\prime }$ denotes the peak value after the FFT.
*Step 7*: Eliminate the estimated *p*-th CPS from the original signal $s_{i}\left(t_{m}\right)$
  (21) $$s_{i}\left(t_{m}\right)={\mathrm{FFT}}_{t_{m}}\left(s_{i}\left(t_{m}\right)\exp\left[j2\pi \left(-\frac{\phi_{2,p}^{\prime }}{2}t_{m}^{2}-\frac{\phi_{3,p}^{\prime }}{6}t_{m}^{3}\right)\right]\right){\mathrm{win}}_{p}\left(f_{t_{m}}\right)\exp\left[j2\pi \left(\frac{\phi_{2,p}^{\prime }}{2}t_{m}^{2}+\frac{\phi_{3,p}^{\prime }}{6}t_{m}^{3}\right)\right]$$
  where ${\mathrm{win}}_{p}\left(f_{t_{m}}\right)=\{\begin{array}{c} 0,\phi_{1,p}^{\prime }-d\leq f_{t_{m}}\leq \phi_{1,p}^{\prime }+d\\ 1,else \end{array}$ denotes the narrowband filter with the *bandwidth* 2d *(can be determined based on the resolution).*
*Step 8*: Repeat Steps 5–7 until the residual signal energy E of the *i*-th range cell is less than $E_{H}$ (saying 5% of the original signal [18,19]), which is an energy threshold.
*Step 9*: If i<I, set *i* = *i* +1 and repeat Steps 3–8 until *i* = I.

**Figure 6:**
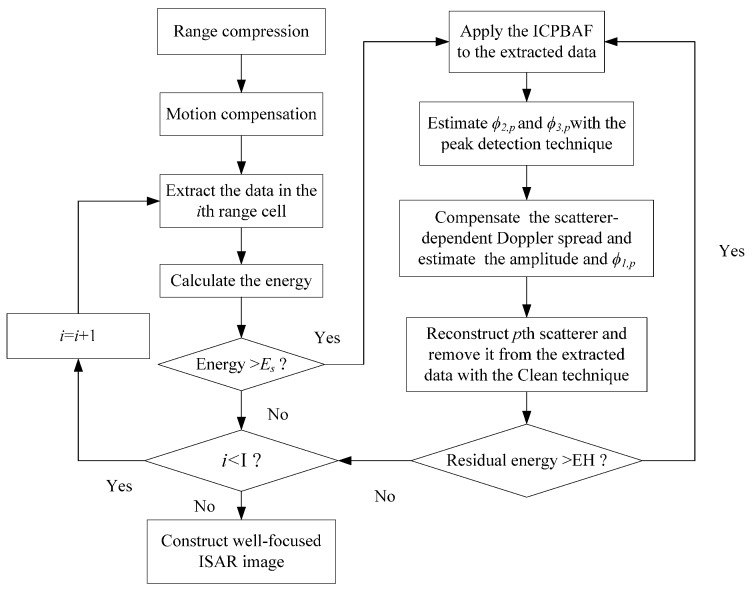
Flowchart of the ICPBAF-based RID ISAR imaging algorithm.

Above is the proposed RID ISAR imaging algorithm for the ship target based on the ICPBAF. With this algorithm, we can construct a well-focused ISAR image for the ship target. In order to provide insight into the working of the RID ISAR imaging algorithm, the flow chart of the proposed RID ISAR imaging algorithm is shown in Figure 6.

## 6. Verification of the ICPBAF-Based RID ISAR Imaging Algorithm

Section 4 verifies high performances of the ICPBAF and Section 5 presents a RID ISAR imaging algorithm based on the ICPBAF. In this section, we employ the synthetic model (Section 6.1) and real radar data (Section 6.2) to verify the superiority and practicability of the ICPBAF-based RID ISAR imaging algorithm.

### 6.1. Verification with the Synthetic Model

**Figure 7:**
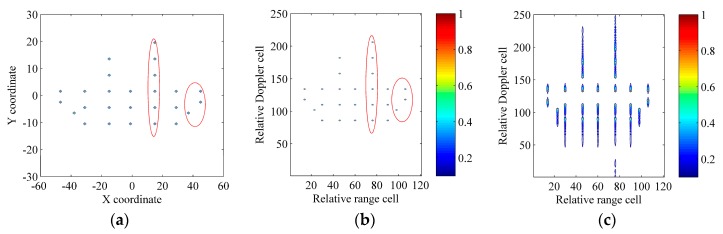
Ship target model and results of the standard RD technique. (**a**) Ship target model; (**b**) Result of the standard RD technique under the absence of the scatterer-dependent Doppler spread; (**c**) Result of the standard RD technique under the existence of the scatterer-dependent Doppler spread.

In this section, referring to [3,8,9,17,18,19,20,21,22], we model the ship target shown in Figure 7a as a set of ideal scatterers. Table 2 gives radar and motion parameters. After the motion compensation, Figure 7b shows the image constructed with the standard RD technique under the absence of the scatterer-dependent Doppler spread, and Figure 7c shows the image constructed with the standard RD technique under the existence of the scatterer-dependent Doppler spread. Obviously, due to the scatterer-dependent Doppler spread, the image quality is degraded in Figure 7c. Note that, as described in Section 1, this paper focuses on the scatterer-dependent Doppler spread compensation and we can refer to [5,6,7] for more details about the motion compensation.

**Figure 8:**
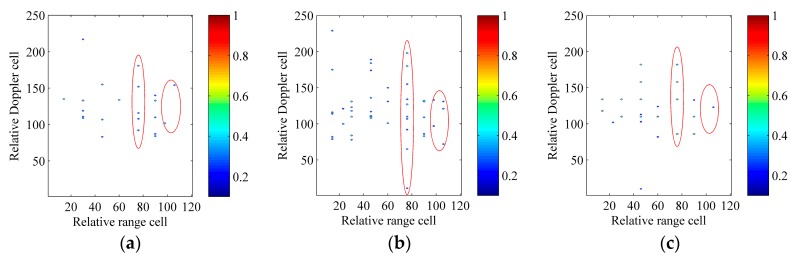

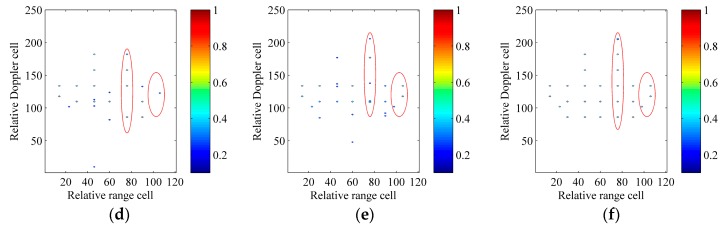
ISAR imaging results of the synthetic model. (**a**) RID ISAR imaging algorithm using the CPF; (**b**) RID ISAR imaging algorithm using the IGCPF; (**c**) RID ISAR imaging algorithm using the SCFT-based algorithm; (**d**) RID ISAR imaging algorithm using the GSCFT-based algorithm; (**e**) RID ISAR imaging algorithm using the GDT-based algorithm; (**f**) RID ISAR imaging algorithm using the ICPBAF.

We contaminate echoes of the ship target with the additive complex white Gaussian noise and the SNR_in_ equals to −5 dB after the motion compensation. Here, RID ISAR imaging algorithms, which use the CPF, IGCPF, SCFT-based algorithm, GSCFT-based algorithm and GDT-based algorithm, are adopted to compare with the ICPBAF-based RID ISAR imaging algorithm. Below, images constructed with these six RID ISAR imaging algorithms are normalized and shown in Figure 8. The entropy of (22) is used as a criterion to measure the quality of the image X(i,n) in Table 3 [12]:

(22) $$ENT=-\sum_{i=1}^{I}\sum_{n=1}^{N_{t_{m}}}{\left|X\left(i,n\right)\right|}^{2}\ln{\left|X\left(i,n\right)\right|}^{2}$$

The superiority of the ICPBAF-based RID ISAR imaging algorithm is obvious in Figure 8. All scatterers are reconstructed correctly and very few spurious scatterers appear. This is because the ICPBAF has the highest cross term suppression and anti-noise performance, which has been analyzed and demonstrated in Section 4. The better focused ISAR image results in the smaller entropy [12,13,18,19,20,21,22]. Results in Table 3 demonstrate the high quality of Figure 8f and validate the superiority of the ICPBAF-based RID ISAR imaging algorithm also.

**Figure 9:**
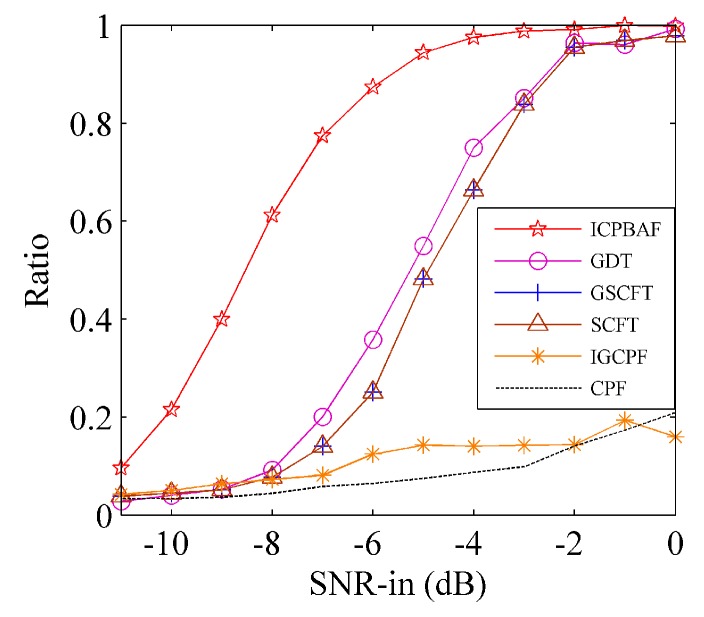
Ratio between the number of the correctly reconstructed scatterers and the number of all reconstructed scatterers.

Actually, the additive complex white Gaussian noise is random. Thus, only with the experiment above, it may not be persuasive to determine the superiority of the ICPBAF-based RID ISAR imaging algorithm. It is known that the result of the Monte Carlo experiment has the generality and representability. Thus, here, a Monte Carlo experiment is used to determine the superiority of the ICPBAF-based RID ISAR imaging algorithm. The data in the 15th range cell, which exists six scatterers and is marked with the red ellipse in Figure 7a, is extracted. We contaminate the extracted data with the additive complex white Gaussian noise. The tested input SNRs are SNR_in_ = [−11:1:0] and 100 trials are performed for each SNR_in_. Aforementioned six RID ISAR imaging algorithms are adopted to perform on the extracted data. In a well-focused ISAR image, most scatterers can be reconstructed correctly and fewest artifacts appear. Thus, the ratio between the number of the correctly reconstructed scatterers and the number of all reconstructed scatterers is used as a measure. Figure 9 shows the simulation result.

The high cross term suppression and anti-noise performance of the ICPBAF guarantee the superiority of the ICPBAF-based RID ISAR imaging algorithm. In Figure 9, even under lower SNRs, the ICPBAF-based RID ISAR imaging algorithm can reconstruct most scatterers correctly and fewest artifacts appear. For example, when SNR_in_ = [−7 dB, −6 dB, −5 dB], the ratio equals to [0.7747, 0.8739, 0.944], which means that the average number of the artifacts is smaller than [1.7449, 0.8658, 0.3559]. This experiment further demonstrates that, compared to RID ISAR imaging algorithms using the CPF, IGCPF, SCFT-based algorithm, GSCFT-based algorithm and GDT-based algorithm, the ICPBAF-based RID ISAR imaging algorithm is more suitable for realistic applications.

### 6.2. Verification with Real Radar Data

**Figure 10:**
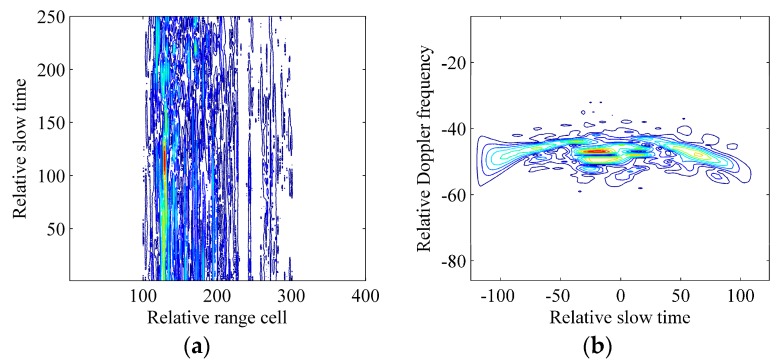
Processing results of the real radar data. (**a**) Azimuth echoes after the motion compensation; (**b**) Wigner-Ville distribution of the 191th range cell.

The real radar data used here is received by a shore-based radar, which works in Ku band with a bandwidth of 240 MHz and a PRF of 125 Hz. The imaged ship target is moving away from the shore-based radar with a velocity of about 10 m/s in the harsh ocean environment. In the real radar data, the number of echo pulses $N_{t_{m}}$ is 250 and the number of the slant range cells is 400. Figure 10a gives the result after the motion compensation with the standard range alignment method, keystone transform and phase gradient autofocus method. The Wigner-Ville distribution of the 191th range cell is given in Figure 10b. According to analyses and simulations in [12,13,21,22], the curve in Figure 10b demonstrates that azimuth echoes of the ship target should be modeled as noisy multicomponent CPSs.

**Figure 11:**
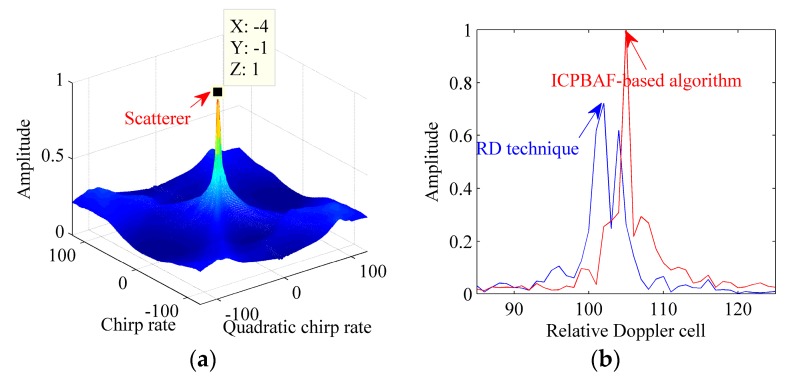
Processing results of the extracted data in the 191th range cell. (**a**) ICPBAF of the extracted data; (**b**) Results of the standard RD technique and ICPBAF-based RID ISAR imaging algorithm.

In order to give an unequivocal evidence that the ICPBAF-based RID ISAR imaging algorithm benefits the imaging quality, we use it to process the extracted data in the 191th range cell. Figure 11 gives the simulation results. The signal energy is accumulated in Figure 11a. With the peak detection technique, the chirp rate and quadratic chirp rate are estimated as −1 Hz/s and −8 Hz/s^2^, respectively. By compensating the Doppler spread pertaining to the estimated parameters and performing an FFT, we complete the energy accumulation in Figure 11b, where the result of the standard RD technique is also shown. Obviously, due to the Doppler spread, the standard RD technique cannot focus the signal energy into the correct Doppler cell. Actually, several scatterers may exist in this range cell. Thus, combing with the Clean technique, we still need to relocate other potential scatterers of this range cell.

**Figure 12:**
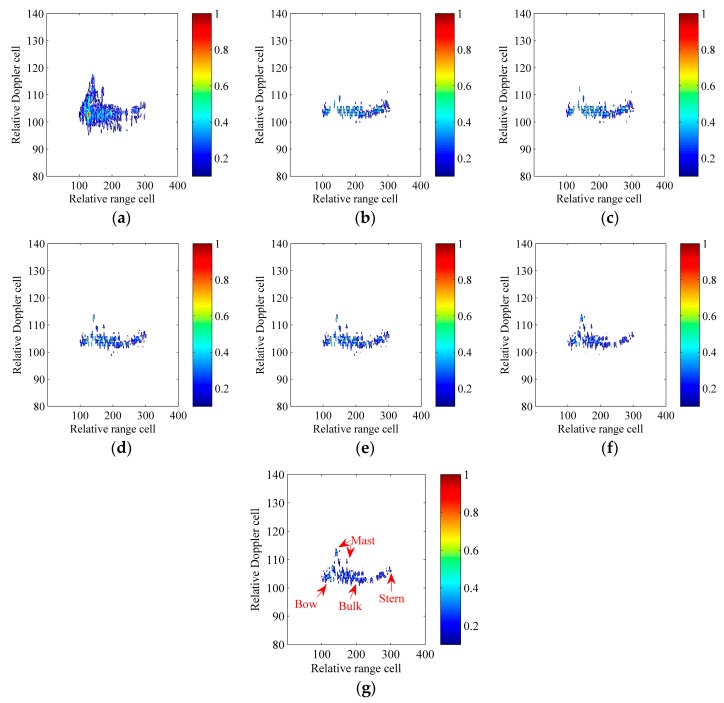
ISAR imaging results of the real radar data. (**a**) Standard RD technique; (**b**) RID ISAR imaging algorithm using the CPF; (**c**) RID ISAR imaging algorithm using the IGCPF; (**d**) RID ISAR imaging algorithm using the SCFT-based algorithm; (**e**) RID ISAR imaging algorithm using the GSCFT-based algorithm; (**f**) RID ISAR imaging algorithm using the GDT-based algorithm; (**g**) RID ISAR imaging algorithm using the ICPBAF.

In Figure 12, RID ISAR imaging algorithms, which use the CPF, IGCPF, SCFT-based algorithm, GSCFT-based algorithm and GDT-based algorithm, are adopted to compare with the ICPBAF-based RID ISAR imaging algorithm. The result of the standard RD technique is also shown. These images are normalized and corresponding entropies are given in Table 4.

Figure 12a is the result of the standard RD technique. Obviously, due to the Doppler spread induced by the chirp rate and quadratic chirp rate, it cannot construct a well-focused image for the ship target. The CPF, IGCPF, SCFT-based algorithm, GSCFT-based algorithm, GDT-based algorithm and ICPBAF can estimate parameters of noisy multicomponent CPSs. Thus, images shown in Figure 12b–g are better than the image shown in Figure 12a, which can also be demonstrated with the entropies listed in Table 4. In Figure 12d–g, it is easy to find that, compared to the SCFT-based algorithm, GSCFT-based algorithm and GDT-based algorithm, the advantage of the ICPBAF-based algorithm is not so obvious. This is because the realistic environment is unknown and we cannot control characteristics of the real radar data, such as the SNR and the number of scatterers in each range cell. Actually, with simulations and analyses in Section 4 and Section 6.1, we know that, if a harsher realistic environment is considered, the advantage of the ICPBAF-based algorithm will be much more obvious. Images shown in Figure 12 and entropies listed in Table 4 verify the practicability of the ICPBAF-based RID ISAR imaging algorithm.

## 7. Conclusions

In this paper, a bilinear coherent estimation algorithm, known as the ICPBAF, is proposed for the CPS. The bilinear ICPBAF can complete the two-dimensionally coherent energy accumulation. The principle, cross term characteristic, computational cost, cross term suppression and anti-noise performance are analyzed for the ICPBAF. Comparisons with five other representative estimation algorithms demonstrate that the ICPBAF can acquire the higher cross term suppression and anti-noise performance with a moderate computational cost. It is worthwhile noting that, for the CPS, the ICPBAF can be seen as the first bilinear coherent algorithm, which has a high practicability. Thereafter, the ICPBAF-based RID ISAR imaging algorithm is presented for ship targets, and we use the synthetic model and real radar data to verify its effectiveness.

## Figures and Tables

**Table 1:** Computational cost.

Algorithm	CPF	IGCPF	SCFT-Based Algorithm
Computational cost	$O\left(N_{t_{m}}^{2}\right)$	$O\left(N_{t_{m}}^{3}\right)$	$O\left(N_{t_{m}}^{3}\right)$
Algorithm	GSCFT-based algorithm	GDT-based algorithm	ICPBAF
Computational cost	$O\left(N_{t_{m}}^{2}\log_{2}N_{t_{m}}\right)$	$O\left(N_{t_{m}}^{2}\log_{2}N_{t_{m}}\right)$	$O\left(N_{t_{m}}^{2}\log_{2}N_{t_{m}}\right)$

**Table 2:** Radar and motion parameters.

Carrier Frequency	15 GHz	Wave Length	0.02 m
Bandwidth	100 MHz	Fast time sampling frequency	200 MHz
PRF	125 Hz	Echo pulses	250
Translational motions	Velocity	Acceleration	Acceleration rate
	24 m/s	2 m/s^2^	1 m/s^3^
Effective rotational motions	Angular velocity	acceleration	acceleration rate
	0.01 rad/s	0.005 rad/s^2^	0.01 rad/s^3^

**Table 3:** Entropies of ISAR images in Figure 8.

	Figure 8a (CPF)	Figure 8b (IGCPF)	Figure 8c (SCFT)
Entropies	7.262	10.1713	5.2378
	**Figure 8d (GSCFT)**	**Figure 8e (GDT)**	**Figure 8f (ICPBAF)**
Entropies	5.2378	4.9776	4.5383

**Table 4:** Entropies OF ISAR images in Figure 12.

	Figure 12a (Standard RD)	Figure 12b (CPF)	Figure 12c (IGCPF)	Figure 12d (SCFT)
Entropies	195.5657	72.0199	72.1156	61.9358
	**Figure 12e (GSCFT)**	**Figure 12f (GDT)**	**Figure 12g (ICPBAF)**	
Entropies	61.9358	60.3322	57.4393	
